# Systematic Review and Critical Appraisal of Cauda Equina Syndrome Management During Pregnancy

**DOI:** 10.7759/cureus.63550

**Published:** 2024-06-30

**Authors:** Chinedu Egu, Samuel Akintunde, Motunrayo Adekoya, Edidiong Essiet, Spyridon Komaitis, Elie Najjar

**Affiliations:** 1 Centre for Spinal Studies and Surgery, Queens Medical Centre, Nottingham University Hospitals National Health Service (NHS) Trust, Nottingham, GBR

**Keywords:** spinal surgery, foetal outcome, lumbar disc herniation, cauda equina syndrome, pregnancy

## Abstract

Cauda equina during pregnancy represents a rare entity, with data regarding optimal treatment being very scarce in the pertinent literature. Given the scarcity of current evidence on the topic, this study conducts a systematic review and analysis of existing literature concerning cauda equina syndrome (CES) management in pregnant women. A comprehensive search was performed across multiple databases, yielding 26 level IV peer-reviewed articles that met the inclusion criteria. These studies collectively encompassed 30 pregnant patients with CES, with a mean age of 31.2 years and an average gestational age of 26 weeks. Disc herniation emerged as the primary cause in 73% of cases. Regarding surgical interventions, the prone position was utilised in 70% of cases, with 73% receiving general anaesthesia. Notably, third-trimester spinal surgeries exhibited a higher complete recovery rate compared to earlier trimesters. Minimally invasive spinal surgery demonstrated superior outcomes in terms of complete recovery and reduced risk of persistent post-operative symptoms when compared to open approaches. Moreover, patients undergoing caesarean section (CS) after spinal surgery reported higher rates of symptom resolution and lower symptom persistence compared to those with CS before spinal surgery or vaginal delivery post-spinal surgery. Despite these study's findings, the overall evidence base remains limited, precluding definitive conclusions. Consequently, the study underscores the importance of multidisciplinary team discussions to formulate optimal treatment strategies for pregnant individuals presenting with CES. This highlights a critical need for further research to expand the knowledge base and improve the guidance available for managing CES in pregnant populations.

## Introduction and background

Cauda equina syndrome (CES) is a spinal surgical emergency, most often attributable to compression of the cauda equina roots and disruption of neuronic signal transmission. It is characterised by a variable constellation of symptoms and signs, including severe low back pain (LBP), radiculopathy, reduced reflexes, saddle anaesthesia, urinary and/or bowel incontinence, and sexual dysfunction [1,2]. A high index of suspicion is essential in diagnosing CES during pregnancy, as many of the common symptoms of pregnancy itself, including urinary dysfunction and back pain, may mimic spinal conditions [3].

CES is rare, with an incidence of 1-3 per 100,000 people [4], accounting for 1-2% of those undergoing surgery for lumbar disc herniation (LDH) [4], and even rarer in pregnancy. It is postulated that hormonal changes, in particular serum relaxin, a hormone that regulates collagen and softens the ligaments of the pelvis in preparation for parturition, may predispose to disc herniation during this time [5]. LDH is the most common cause of CES, with other causes including spinal lesions or tumours, lumbar spinal stenosis, spinal infections, lower back trauma, spinal arteriovenous malformations, spinal haemorrhage, spinal ischemic insults, or post-operative spinal surgery complications [6].

Opting for surgical intervention during pregnancy must always balance the risks and benefits of treatment for both the mother and foetus. Hence, clinical decision-making must be meticulous and ideally made by a multidisciplinary team (MDT) of anaesthetists, neonatologists, obstetricians, and surgeons [7]. The effects of patient positioning, anaesthesia, foetal monitoring, plans for urgent delivery, and monitoring maternal blood pressure may have a detrimental effect on the outcome and must be carefully weighted [8].

There is limited evidence available for the optimal management options for CES in pregnancy. Most of the evidence derives from case reports and series, and no randomised controlled data is available, resulting in contradictory recommendations. With the above in mind, the overreaching goal of this study was to systematically review the current evidence for the management of CES in pregnancy and analyse the available literature. To our knowledge, this is the first systematic review focusing on the management of CES during pregnancy. This article was previously presented as an oral presentation at the 2023 British Association of Spinal Surgeons (BASS) annual scientific meeting on April 20, 2023.

## Review

Materials and methods

The Preferred Reporting Items for Systematic Reviews and Meta-Analyses (PRISMA) guidelines were followed [9]. A literature search was conducted using five databases (Medline, Embase, PubMed, Cochrane Library, and Science Direct) from 1946 to February 2022. The inclusion of studies from such an extended timeframe is justified by the very limited availability of data on the topic. The search strategy used a combination of keywords and Medical Subject Headings, which is shown in Table 1.

**Table 1:** Search Strategy

#1	("Caud* Equin* Syn*" or "Intervertebral Dis* Displacement" or "symptomatic lumbar dis* herni*" or "disc herni*" or "caud* equin* compres*" or "dis* prolap*" or "dis* rupture" or "dis* perfor*" or "nucleus pulposus herni*" or "spin* stenos*" or "spin* lesion*" or "spin* malig*")
#2	("gravid*" or "child bearing" or "parturien*" or "pregna*" or "Pregnancy")
#3	(interven* or treat* or manag* or therap*)
#4	#1 AND #2 AND #3

All studies that evaluated any intervention for treating CES during pregnancy were eligible for inclusion. In light of the limited clinical evidence, case reports and case series were included. The methodology used was in line with the recommendations of the Cochrane Back Review Group [10].

Inclusion and Exclusion Criteria

We included studies reporting CES during pregnancy, including symptoms, radiographic findings, management details, and outcomes related to surgical intervention and foetal outcomes. We excluded articles in languages other than English, those focused on the prevention of CES and not a treatment, and also where interventions were started before pregnancy but measured symptoms during pregnancy. Furthermore, we excluded articles with participants with the onset of symptoms during labour and conference abstracts and reviews (due to data duplication).

Identification of Studies

Studies identified by the search strategy were assessed for inclusion. Duplicates were removed, and the remaining studies were screened using their titles, abstracts, and full texts. The process was performed independently by two researchers, and in cases of disagreement, the studies were discussed with the supervising researcher to reach a consensus.

Quality Assessment

A quality assessment was carried out using the Joanna Briggs Institute Critical Appraisal Checklist for Case Reports [11]. This consists of eight questions accounting for clear documentation of the patient’s demographics, history, presentation of clinical condition, the diagnostic tests used and their results, the intervention used, adverse events, and the clinical outcome.

Data Extraction

A narrative synthesis of the findings from the included studies was conducted due to the heterogeneity of the studies and their results, including differences in design, outcome, and measure of effect. Hence, the information was pooled into subgroups for analysis.

The type of surgical intervention, types of anaesthetics, and patient positioning during surgery were included. All studies had clinical and functional outcome measures evaluated at least once following intervention during pregnancy.

Primary Outcomes

Primary outcomes include pain intensity, neurological deficits, sensory changes or loss of motor function, bowel or urinary symptoms, foetal outcomes, and surgical complications, including infection, wound dehiscence, re-herniation, and re-operation. A meta-analysis was not conducted, mainly due to the significant heterogeneity of the available data.

Data Analysis

The results were analysed narratively in subgroups, comparing the time of intervention (by trimester) with regards to maternal and foetal outcomes. We further explored the spinal approaches, mode of delivery, and time to surgery regarding maternal and foetal outcomes. Arithmetic means and percentages were calculated for comparisons between each sub-group.

Results

**Figure 1:**
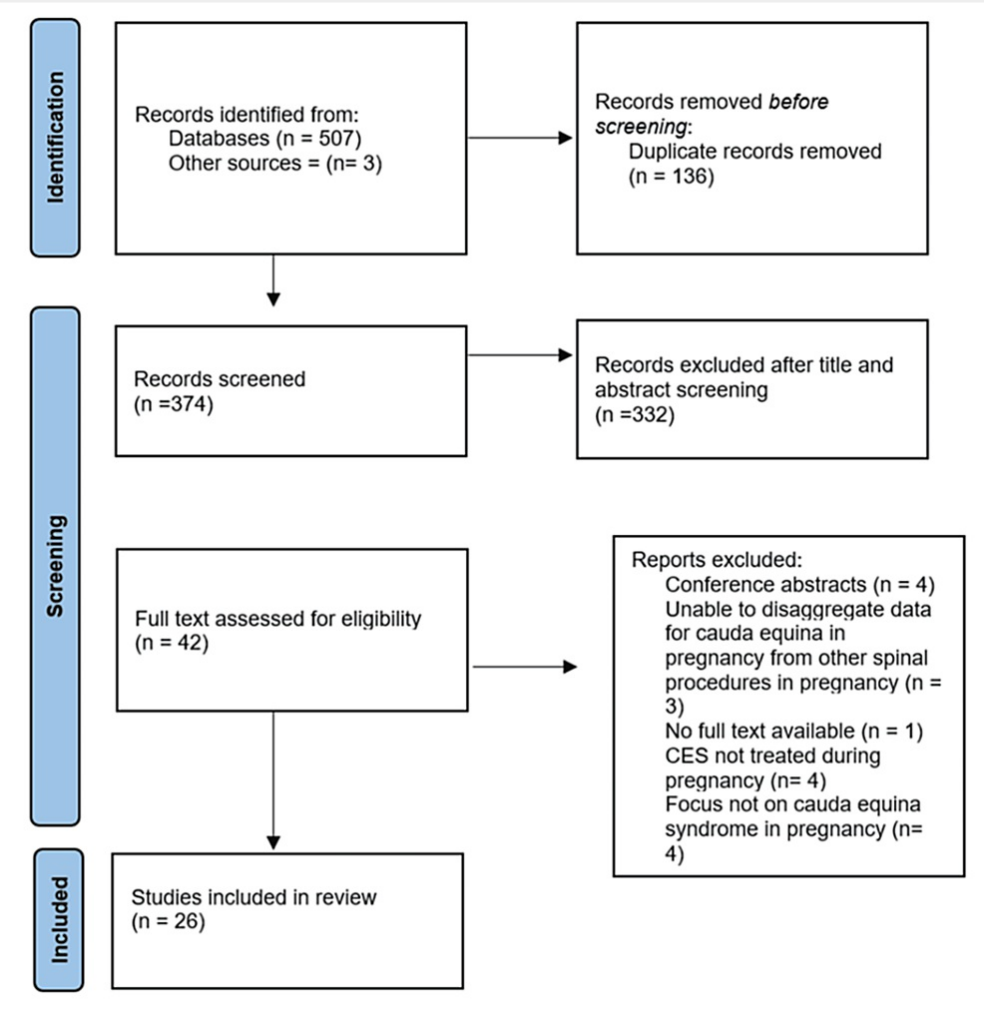
PRISMA flowchart illustrating the included and excluded studies PRISMA: Preferred Reporting Items for Systematic Reviews and Meta-Analyses

A systematic search of Medline, Embase, PubMed, Cochrane Library, and Science Direct identified 507 articles. An additional three articles were found through the reference lists of these studies. One hundred and thirty-six duplicates were removed, leaving 374 articles to be screened. Of these, 332 records were excluded at the title and abstract stages due to non-fulfilment of the inclusion criteria. The remaining 42 articles were screened using the full text, and a further 16 articles were excluded, leaving 26 studies (all level IV) to be used in the qualitative synthesis (Figure 1).

Participants' Demographics

From the included 26 studies, data were extracted from 30 pregnant women and used in this review. The participants' ages in the review ranged from 20 to 41 years, with a mean of 31.2 years (SD=5.2). The mean age of patients in their first trimester (n=2) was 38 years (SD=1.4). The second trimester (n=15) had a mean age of 31 years (SD=5.2), and the third trimester (n=13) participants were 30.3 years old (SD=4.9). The included patient’s gestational age ranged from 11 to 36 weeks, with a mean of 26 weeks (SD=7.3). The mean gestational age of each trimester includes the first trimester, 11.5 weeks (SD=0.7); the second trimester, 22 weeks (SD=3.9); and the third trimester, 33 weeks (SD=2.5). The proportion of included patients with single pregnancies was 90% (n=27), twin gestation was 6.7% (n = 2), and one patient had a triplet. The proportion of patients who have only been pregnant once was 40% (n=12), those between two and five times was 36.7% (n=11), and only one patient had more than five previous pregnancies, while no information was obtained from six patients. Significant past medical history was obtained from 14 patients; 10 had chronic lower back pain, of which two had confirmed degenerative spine disease. Three patients had a high BMI, with chronic lower back pain in two of these patients. One patient had previous spine surgery, and one was diabetic.

The reported symptoms included LBP in 90% (n=27), urinary retention/incontinence in 80% (n=24), saddle hypoesthesia in 63.3% (n=20), leg pain (unilateral; 43.3%, n=13, bilateral; 33.3%, n=10), bowel constipation/incontinence in 60% (n = 12), and lower limb weakness (unilateral; 33.3%, n=10, bilateral; 13.3%, n=4). Neurological examination findings included saddle hypoesthesia (to pinprick; 66.6% n=20, to light touch; 60% n=18), reduced limb power (unilateral; 36.7% n=11, bilateral; 20% n=6), abnormal anal tone 56.7% (n=17), reduced reflexes 30% (n=9), and positive SLR in 26.7% (n=8). The mean post-void bladder scan volume was 541mls (SD=313).

MRI of the spine was the investigation of choice for 93% (n=28) of the patients. One patient harbouring a spinal hemangioblastoma causing CES underwent an initial CT scan without contrast followed by an MRI of the lumbar spine, while no information was provided on the imaging modality used to diagnose one patient with disc herniation.

**Figure 2:**
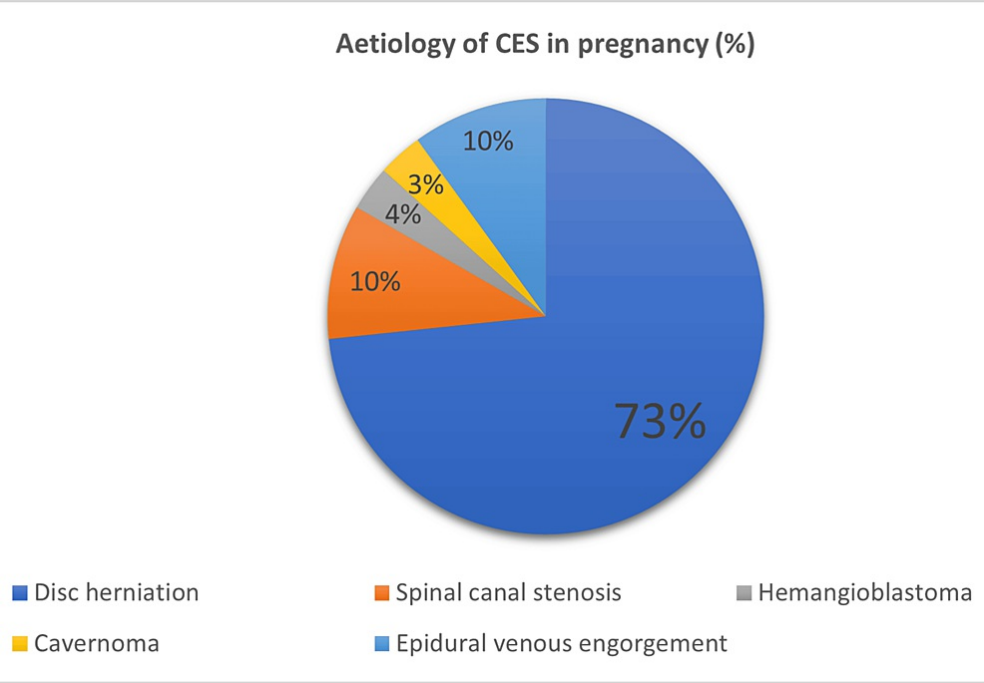
Aetiology of CES in pregnancy CES: cauda equina syndrome

As stated above, the most common cause of CES in pregnancy was disc herniation (73.3%, n=22). There were three cases each of spinal canal stenosis and epidural venous engorgement. One case of cavernoma and hemangioblastoma was identified in Figure 2. Single-spinal-level pathology was identified in 73.3% (n=22), with the L5-S1 (46.6% (n=14)) level being the most common culprit.

Intervention

MDT discussions involving at least an obstetrician, neonatologist, anaesthetist, and spinal surgeon were undertaken in 73.3% (n=22) of the cases. No information about MDT was given to 26.7% (n=8). Spinal surgical intervention was undertaken to relieve the CES symptoms in 96.7% (n=29) of patients. The only case that was not selected for spinal surgery was diagnosed with epidural venous engorgement secondary to increased intra-abdominal pressure, with symptoms improving following a caesarean section (CS).

Most spinal surgeries were via the open approach (73.3%, n=22), and others were through minimally invasive/endoscopic approaches (13.4%, n=7). Decompressive surgery was carried out within 48 hours in 83.3% (n=25) of cases, while 16.7% (n=5) had surgery outside the 48-hour window. Most of the patients (70%, n=21) were operated on in a prone position, while two patients were positioned in the left and right lateral positions, respectively. General anaesthesia (GA) was provided in 73.3% (n=22) while spinal anaesthesia was used in 13.4% (n=4). The mean length of surgery was 153 minutes (SD=59), ranging from 60 to 240 minutes.

**Figure 3:**
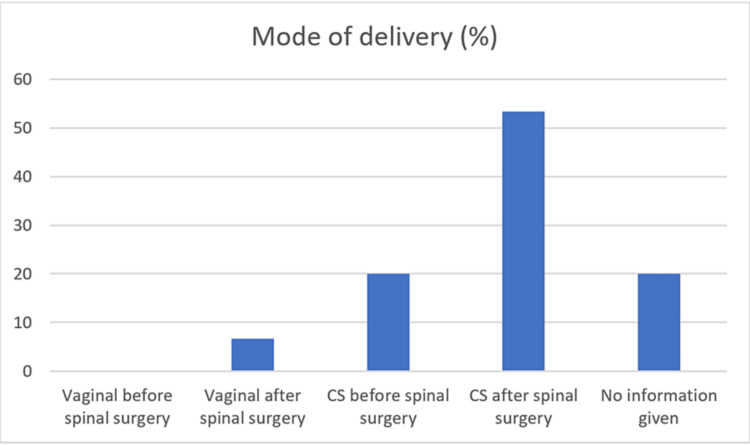
Details of the mode of delivery CS: caesarean section

In 53.3% (n=16) of patients, it was decided to proceed with a CS after spinal surgery, while two cases had vaginal delivery after spinal surgery. Six patients had CS before their spinal surgery, and no patient had a vaginal delivery prior to spinal intervention (Figure 3). The mode of delivery wasn’t specified in six of the cases. The average time to delivery after spinal surgery was 8.7 weeks (SD=8.6), ranging from 0 to 28 weeks.

Foetal Monitoring

Intra-operative foetal monitoring has been used in 23.3% (n=7), and two studies reported monitoring the foetus both in the pre- and post-operative period. In two cases, the foetus has been assessed only post-operatively, and in one case, only pre-operatively. No information on foetal monitoring was provided in 17 studies. In terms of monitoring modality, foetal Doppler ultrasound was used for foetal assessment in 13.3% (n=4) of the cases. Foetal heart rate (FHR) monitoring was used in three cases, while others either performed foetal ultrasound imaging (6.7%, n=2) or a cardiotocogram (3.3%, n=1).

Follow-Up and Bias

**Figure 4:**
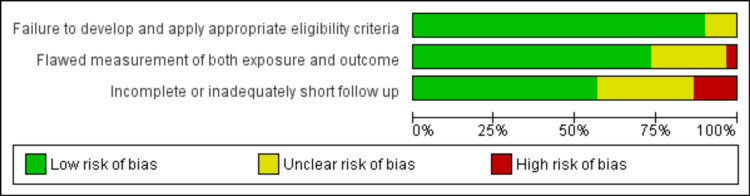
Risk of bias graph: review authors' judgements about each risk of bias item presented as percentages across all included studies

**Figure 5:**
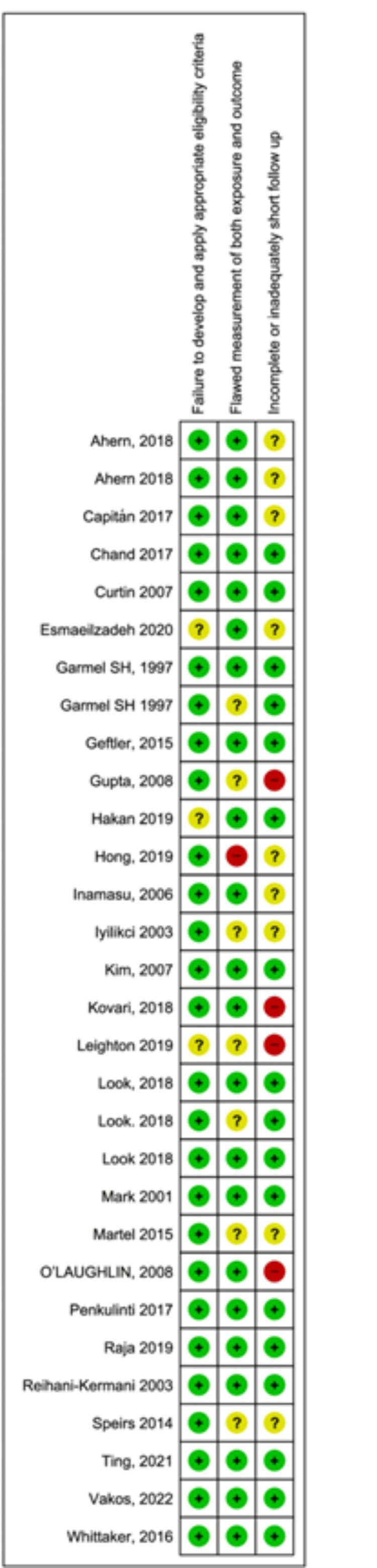
Risk of bias summary showing review authors' judgements about each risk of bias item for each included study

There was adequate follow-up (greater than six weeks) in 19/30 patients (63%). The included studies were all either case reports or case series, which increased their risk of bias and limited the methodological quality. A summary assessing the risk of bias for individual studies was constructed to depict the measurable influences (Figures 4-5).

Measurement of Exposure and Outcome

Sufficient information was provided concerning the intervention used and the maternal and infant outcomes in 22/30 (73%). Seven of 30 patients (23%) had some missing data, and 1/30 (4%) had insufficient data.

Outcome

**Figure 6:**
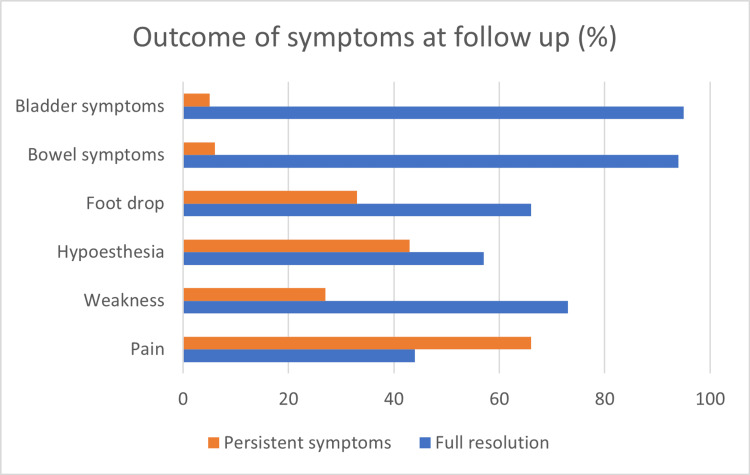
Summary of patient-reported outcomes at follow-up

All studies in this review included information on the outcome of managing CES in pregnancy (Figure 6 and Table 2). The outcomes were analysed based on the trimester of spinal surgery. Among the cohort, two patients had spinal surgery in their first trimester. Their surgeries were performed within 48 hours of symptom onset, and they both had persisting symptoms at follow-up. In cases operated on in the second trimester, 80% (n=12) were operated on within 48 hours of symptom onset; others were later. They reported complete resolution of symptoms in 33.3% (n=5) and persistence of either weakness, hypoesthesia, paraesthesia, bowel, or bladder symptoms in 66.7% (n=10). Among the population of patients operated on during the third trimester, 85% (n=11) had their operation within 48 hours, while 15% (n=2) were delayed (>48 hours). Five cases (41.7%) reported full resolution of symptoms, whereas symptoms that persisted at follow-up were seen in 58.3% (n=7).

**Table 2:** Details of radiological findings, patient positioning, mode of anaesthesia, surgical approach, symptoms at follow-up, and foetal outcome GA: general anaesthesia

Trimester of spinal surgery	Radiological findings	Patient positioning	Mode of anaesthesia	Open or minimally invasive spinal surgery	Symptoms at follow-up	Outcome of infant
1	Disc herniation	No information given	No information given	Open	Persistent	Healthy infant
1	Disc herniation	Prone	GA	Minimally invasive	Persistent	Healthy infant
2	Disc herniation	Prone	GA	Open	Persistent	No information given
2	Disc herniation	Prone	GA	Open	Persistent	Miscarriage
2	Disc herniation	Prone	GA	Open	Resolved	Healthy infant
2	Cavernoma	Prone	GA	Open	Persistent	Healthy infant
2	Disc herniation	Prone	Epidural	Open	Persistent	Healthy infant
2	Disc herniation	Prone	GA	Minimally invasive	Resolved	Healthy infant
2	Epidural venous engorgement	Prone	GA	Open	Persistent	no information given
2	Spinal canal stenosis	Prone	No information given	Open	Persistent	Healthy infant
2	Disc herniation	Left lateral	GA	Open	Resolved	Healthy infant
2	Disc herniation	Prone	GA	Minimally invasive	Resolved	Healthy infant
2	Disc herniation	Prone	GA	Minimally invasive	Resolved	No information given
2	Disc herniation	Prone	No information given	Open	Persistent	Healthy infant
2	Disc herniation	Prone	GA	Minimally invasive	Persistent	Healthy infant
2	Disc herniation	No information given	Epidural	Open	Persistent	No information given
2	Disc herniation	Prone	GA	Open	Persistent	Healthy infant
3	Disc herniation	Left lateral	GA	Open	Resolved	Healthy infant
3	Epidural venous engorgement	Prone	GA	Open	Persistent	Healthy infant
3	Disc herniation	Right lateral	Spinal	Minimally invasive	Persistent	Healthy infant
3	Hemangioblastoma	Prone	GA	Open	Persistent	Healthy infant
3	Disc herniation	Prone	GA	Open	Persistent	no information given
3	Disc herniation	Prone	GA	Open	Persistent	Healthy infant
3	Epidural venous engorgement	No information given	Spinal	No information given	Resolved	Healthy infant
3	Disc herniation	Prone	GA	Open	Persistent	Healthy infant
3	Spinal canal stenosis	No information given	No information given	Open	Persistent	Healthy infant
3	Disc herniation	Prone	GA	Open	Resolved	Healthy infant
3	Disc herniation	No information given	GA	Open	Persistent	Healthy infant
3	Disc herniation	Right lateral	GA	Minimally invasive	Persistent	Healthy infant
3	Spinal canal stenosis	Prone	GA	Open	Resolved	

In comparing the surgical approach, minimally invasive surgery (MIS) was associated with better chances of full resolution of symptoms (50% vs. 23%) and a lower risk of persistent symptoms (50% vs. 77%) compared to the open approach; however, the timing of surgery within 48 hours in both open and MIS was similar, at 80% in both arms. Patients who had their spinal surgery within 48 hours did better in terms of complete resolution of symptoms (32% vs. 20%) and less persistent symptoms (68% vs. 80%) than those falling within the late group (>48 hours).

Participants who had CS after spinal surgery reported a higher rate of full resolution of symptoms (44% vs. 34% vs. 0%) and fewer chances of developing persistent symptoms (56% vs. 66% vs. 100%) compared to those who had CS before spinal surgery and vaginal delivery after spinal surgery, respectively. One patient who had a vaginal delivery after spinal surgery re-herniated four days post-delivery and required re-operation.

Overall, 79% (n=21) of infants were healthy, with the first and third trimesters recording 100% of healthy infants and the second trimester recording 66.7% (n=10), with one patient miscarried after spinal surgery, and no information on foetal outcome was provided in four cases. Patients placed in the right and left lateral positions during spinal surgery reported a 100% successful birth rate with no mother- or infant-related complications. Twenty per cent of patients placed prone didn’t have any information on the foetal outcome, but 75% (n=15) recorded healthy infants and only one miscarriage during the second trimester of pregnancy with healthy mothers. There was no maternal mortality recorded in any of the studies.

Discussion

The demographics, management, and outcomes of 26 case reports or case series involving 30 patients were collated and compared through a systematic review. Overall, patients operated during the third trimester, within 48 hours of presentation, underwent MIS and delivery with CS post-operatively and had higher rates of complete resolution of symptoms at follow-up when compared with their counterparts. There were no statistically significant differences in maternal or foetal outcomes based on the patient's positioning during spinal surgery. Current evidence corroborates the theory that non-obstetric surgery is safe in experienced hands for both mother and foetus [7,12,13].

Clinical Implications and Repercussions

The management of CES during pregnancy should be ideally planned by an experienced MDT, which comprises at least an obstetrician, neonatologist, anaesthetist, and spinal surgeon. In terms of imaging modality, MRI is not contraindicated in pregnancy and can be safely used to investigate CES [5,14-16].

Controversies exist about the timing of surgical decompression, but several studies and meta-analyses report better prognoses for patients who undergo surgery within 48 hours from the beginning of symptoms [17,18]. In our review, the maternal and foetal outcomes were better when spinal surgery was performed within 48 hours.

There is a paucity of literature with regard to ideal positioning. It is well established that spinal surgery in gestational patients can be performed both in prone and lateral positions [13]. A prone patient allows better access; however, the lateral position may prevent abdominal compression [13]. The prone position is not recommended beyond 12 weeks of gestation [13,19] without using the Relton-Hall Laminectomy frame as it can cause abdominal compression, inciting preterm labour [14,20,21].

Pregnancy is not a contraindication for general or regional anaesthesia [5,14,15,22]. Regional anaesthesia is recommended for shorter operations [23]. GA should be used cautiously in the first trimester due to the increased risk of spontaneous abortion [24]. The American College of Obstetricians and Gynaecologists (ACOG) recommends regional anaesthesia for surgery in pregnancy when possible [25].

The options for surgical management of CES range from traditional open procedures to minimally invasive spinal surgery techniques based on aetiology. Spine surgeons consider microdiscectomy the “gold standard” technique for lumbar discectomy as it presents shorter hospital stays and lower complications [26]. In our review, due to the small numbers and heterogeneity of cases, we couldn’t establish a reliable superiority of one approach over the other as the surgical outcome is governed by other factors such as the timing of surgery, whether complete or incomplete CES, and degree of decompression, which were not entirely accounted for by the reviewed articles. Bipolar electrocautery was used to manage epidural venous engorgement causing CES in this review. In the analysed cohort of patients, the only case of a spinal tumour, a cavernoma, underwent a successful CS before tumour resection and hematoma evacuation at 26 weeks gestation. However, the infant died within five days of birth due to ileus and pulmonary insufficiency.

The ACOG recommends FHR measurement using Doppler ultrasound before and after any surgical intervention, regardless of gestational age, with the addition of contraction monitoring in the viable foetus [25]. The Royal College of Obstetrics and Gynaecologists (RCOG) recommends that continuous monitoring is not needed when a woman is healthy and has no significant history of obstetric complications but is recommended if any indications of foetal compromise are present [27]. ACOG further recommended that an MDT determine the use of intraoperative foetal monitoring based on each patient and the surgery to be performed [25].

There is controversy regarding the optimal delivery route for patients who do not undergo a CS during spinal decompression surgery. Some physicians recommend a CS to prevent further spinal-related complications like recurrence due to increased intra-abdominal pressure [28-30]. However, among women with vaginal deliveries, there is no report of an increased rate of persistent neurological symptoms [29]. Brown and Brookfield postulated that labour induction before treatment for LDH can cause increased neurological injury due to increased epidural venous pressure during labour [20]. In our review, women who had CS after spinal surgery had a higher rate of symptom resolution and fewer persistent symptoms compared to the CS before spinal surgery and vaginal birth after spinal surgery patient groups. A higher quality of evidence, MDT-based decisions, and individualised approaches should guide the delivery route. The included studies are summarised in Table 3.

**Table 3:** Study characteristics

S/N	Study ID (last name, year or publication)	Study design	Country where the study was conducted	Level of evidence
1	Curtin and Rice, 2007 [28]	Case report	Ireland	4
2	Antón Capitán and Malillos Torán, 2017 [21]	Case report	Spain	4
3	Penkulinti et al., 2017 [31]	Case report	India	4
4	Ahern et al., 2018 [32]	Case series	Ireland	4
5	Speirs et al., 2014 [33]	Case report	United Kingdom	4
6	Esmaeilzadeh et al., 2020 [34]	Case report	Germany	4
7	Brown et al., 2001 [5]	Case report	USA	4
8	Look, Kleck, and Burger, 2018 [35]	Case series	USA	4
9	Leighton, Townsend, and Leslie, 2019 [36]	Case report	United Kingdom	4
10	Iyilikci et al., 2003 [15]	Case report	Turkey	4
11	Martel, Volpi-Abadie, and Ural, 2015 [37]	Case report	USA	4
12	Raja, Kanna, and Rajasekaran, 2019 [38]	Case report	India	4
13	Chand et al., 2017 [39]	Case report	India	4
14	Hakan, 2012 [40]	Case report	Turkey	4
15	Garmel et al., 1997 [41]	Case series	USA	4
16	Reihani-Kermani, 2003 [16]	Case report	Iran	4
17	Kovari and Horvath, 2018 [42]	Case report	Hungary	4
18	Inamasu, Nichols, and Bernard, 2006 [43]	Case report	USA	4
19	Gupta et al., 2008 [44]	Case report	United Kingdom	4
20	Whittaker and Garcia, 2016 [45]	Case report	USA	4
21	Hong, Theron, and Churchill, 2019 [46]	Case report	United Kingdom	4
22	Geftler et al., 2015 [22]	Case report	Israel	4
23	O'Laughlin and Kokosinski, 2008 [47]	Case report	USA	4
24	Vakos et al., 2022 [48]	Case report	USA	4
25	Kim et al., 2007 [14]	Case report	Korea	4
26	Ting et al., 2021 [49]	Case report	Ireland	4

Limitations

This systematic review incorporates level IV evidence derived from a small number of studies and a limited cohort of patients (26 studies, 30 patients). Studies had poor methodological quality, a high bias rate, a lack of randomisation, and incomplete outcomes. Selection bias was a significant confounder, often preventing reliable conclusions about patient positioning, mode of delivery, and open vs. minimally invasive spinal surgeries.

The reports included women at various stages throughout their pregnancy and with varying symptoms and interventions, using variable quantitative and qualitative data to measure interventions' effects, hence making the data not directly comparable. Follow-up duration was not reported in 11 studies, causing a high risk of attrition bias. Finally, a limited representation of patients’ baseline function prevented comparisons from being made pre- and post-treatment.

## Conclusions

This study offers the first systematic analysis of the literature regarding the management of CES during pregnancy. The management of pregnant women with confirmed CES should involve a multidisciplinary team to devise the most effective treatment strategy. The lack of universal guidelines for managing these patients often results in delayed diagnosis and treatment, increasing the risk of chronic neurological complications. Early identification of the pathology is crucial to achieving better outcomes. Due to the limited available literature, it is possible to offer recommendations but not to draw definitive conclusions about the optimal management of CES in pregnant women.
